# Anchor Link Prediction across Attributed Networks via Network Embedding

**DOI:** 10.3390/e21030254

**Published:** 2019-03-06

**Authors:** Shaokai Wang, Xutao Li, Yunming Ye, Shanshan Feng, Raymond Y. K. Lau, Xiaohui Huang, Xiaolin Du

**Affiliations:** 1 Guanghua School of Management, Peking University, Beijing 100871, China; 2 Harvest Fund Management Co., Ltd., Beijing 100005, China; 3 School of Computer Science and Technology, Harbin Institute of Technology, Shenzhen 518055, China; 4 Tencent, Shenzhen 518057, China; 5 Department of Information Systems, City University of Hong Kong, Kowloon Tong, Hong Kong, China; 6 School of Information Engineering Department, East China Jiaotong University, Nanchang 330013, China; 7 College of Computer Science, Beijing University of Technology, Beijing 100124, China

**Keywords:** anchor link prediction, network embedding, attributed network

## Abstract

Presently, many users are involved in multiple social networks. Identifying the same user in different networks, also known as anchor link prediction, becomes an important problem, which can serve numerous applications, e.g., cross-network recommendation, user profiling, etc. Previous studies mainly use hand-crafted structure features, which, if not carefully designed, may fail to reflect the intrinsic structure regularities. Moreover, most of the methods neglect the attribute information of social networks. In this paper, we propose a novel semi-supervised network-embedding model to address the problem. In the model, each node of the multiple networks is represented by a vector for anchor link prediction, which is learnt with awareness of observed anchor links as semi-supervised information, and topology structure and attributes as input. Experimental results on the real-world data sets demonstrate the superiority of the proposed model compared to state-of-the-art techniques.

## 1. Introduction

In recent years, with the popularity of various social network platforms, a user is usually involved in multiple social networks simultaneously [1]. Due to the function diversity, users in different platforms may express their opinions on various topics, share distinct types of content or follow different users. For example, a user may use Facebook to follow and share entertainment news, and use Quora to gain and share knowledge. The social network platforms profile users from different points of view. If we can identify the same user in different social networks, her profile can be better characterized for a more accurate classification or recommendation. The problem of identification of the same users across multiple networks is known as anchor link prediction, where the associations are termed as anchor links [2].

Despite great application values, solving the problem is challenging, because of the complex network structures, the rich attribute information, and few observed anchor links. Early studies mainly solve the problem by exploiting user profiles (e.g., user name, location, gender) [3,4], demographical features [5] or user generated contents, such as, tweets, posts and reviews [6]. Recently, network structures are also leveraged to address the problem. Particularly, methods of this type rely on hand-crafted network features. For example, match degree can be computed using the number of shared identified friends [7], in/out neighbors and in/out degree [8], and Dice coefficient [9]. However, the hand-crafted features only partially reflect the intrinsic structure regularities of networks, thereby producing less satisfactory performance for anchor link prediction.

Recently, network-embedding techniques have been used to learn latent network features, which can better preserve the structural regularity of a network [10,11]. To match users in different social platforms, Liu et al. [12] proposed IONE. The algorithm learns the embedding of a user by predicting her network contexts, i.e., the follower-ship/followee-ship, where observed anchor links are used to transfer contexts between networks. Man et al. [13] proposed a supervised model PALE, which maps each user into a low dimension space for the identification of anchor links. However, the two models use only the network topology without considering node attributes, which are not applicable to attributed networks. Moreover, in practice the observed anchor links are very few and thus a semi-supervised solution is quite advisable and desirable.

In this paper, we propose a novel semi-supervised model (APAN) to tackle the Anchor lLink Prediction across Attributed Networks. The key idea of APAN is learning the embedding of each network to represent its users into a low-dimensional space. On the one hand, the low-dimensional representation of each user is used to predict her contexts in the network, i.e., the random-walk sequences generated via network topology and the neighboring nodes that share same attribute information. On the other hand, it is leveraged to predict the anchor links (semi-supervised information) between networks. By doing so, the nodes (users) that have similar structure contexts and attributed information will be close in the embedding space. Also, the anchor link predictor is simultaneously trained as the embeddings are learnt. The real-world data sets are used to evaluate the performance of the proposed APAN model. Experimental results show that APAN outperforms state-of-the-art competitors.

## 2. Related Work

Recently, network embedding has aroused a lot of research interest. Network embedding aims to learn low-dimensional representations of network nodes, while effectively preserving network topology structure, node content, and other side information. Inspired by the idea of word representation learning [14], Perozzi el al. [10] developed DeepWalk to learn the representations of nodes in a network, which can preserve the neighbor structures of nodes. Node2vec [15] further exploits a biased random-walk strategy to capture more flexible contextual structure. Network structures include first-order structure and higher-order structure. LINE [11] is proposed to preserve the first-order and second-order proximities. The first-order proximity is the observed pairwise proximity between two nodes. The second-order proximity is determined by the similarity of the neighbors of two nodes. Besides network structures, node content is another important information source for network embedding. With content information incorporated, the learnt node representations are expected to be more informative. Yang et al. [16] propose TADW that takes the rich information (e.g., text) associated with nodes into account when they learn the low-dimensional representations of nodes. Pan et al. [17] propose TriDNR which is a coupled deep model that incorporates network structure, node attributes, and node labels into network embedding. LANE [18] is also proposed to incorporate the label information into the attributed network embedding. The task of linking users accounts on multiple social networks, is a challenging task, because social network structures for a specific user can be rather diverse on different social media platforms. Since the traditional network-embedding methods are designed for single network, they cannot handle the anchor link prediction problem. Moreover, the network embeddings are usually learnt in an unsupervised manner, and hence cannot leverage the observed anchor links in anchor link prediction.

Conventional methods for finding correspondence between networks can be mainly divided into two categories. The first category is called network alignment. It works in an unsupervised manner and does not leverage the existing correspondence. Specifically, the type of methods aligns nodes by finding structural similarity between nodes across networks. Network alignment has been widely used in many fields such as bioinformatics [19], computer vision [20], database matching [21], etc. However, ignoring the observed correspondence is obviously a waste of knowledge. The second category belongs to supervised methods, which learns a predictor relying on the observed anchor links [22]. Most of studies train the predictor directly using the hand-crafted network features, such as common neighbors [7], degree [8], clustering coefficient [9], etc. However, the hand-crafted features may not capture all the intrinsic structural regularities of the networks, thereby producing less satisfactory performance.

With the advancement of deep learning, network-embedding techniques are developed to identify the same users in different platforms. For example, Liu et al. [12] proposed IONE algorithm, which embeds users into a low-dimensional space for anchor link prediction. Man et al. [13] proposed an embedding and matching-based model PALE. However, different from our approach, the network embedding in PALE is purely unsupervised and does not leverage observed anchor links when encoding the network structure into embeddings. Moreover, the two approaches cannot make use of network attributes. Recently, Zhang et al. [23] proposed an attributed network alignment algorithm, called FINAL. The method leverages the node attribute information to guide the topology-based alignment. In FINAL, a nice alignment consistency principle is designed and developed, i.e., the alignments between two pairs of nodes across the networks should be “similar/consistent” with each other. However, this algorithm works in an unsupervised manner and cannot leverage the observed anchor links.

## 3. Methods

### 3.1. Problem Formulation

**Figure 1:**
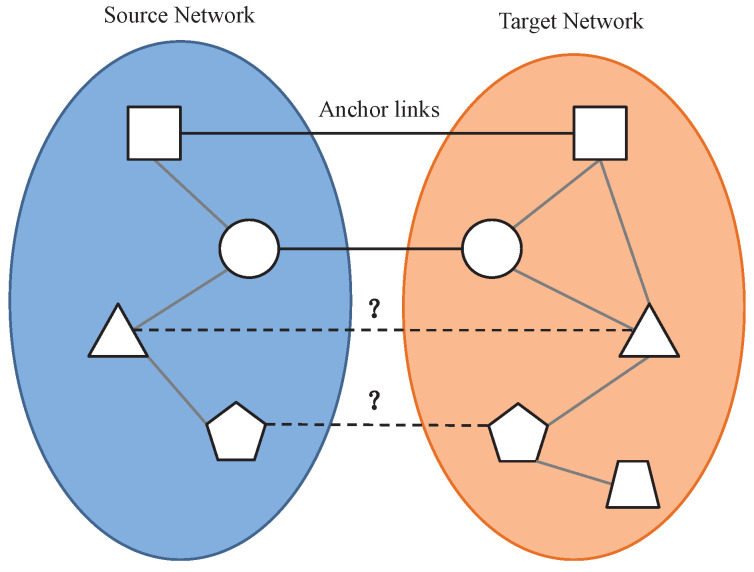
The anchor link prediction problem.

Assume we are given an attributed network G=(X,E,A), where $X=\{x_{1},\ldots ,x_{N}\}$ is a set of nodes, *E* is the adjacent matrix, $E_{ij}$ is the weight of the edge between nodes $x_{i}$ and $x_{j}$. If there is a connection between $x_{i}$ and $x_{j}$, $E_{ij}=1$, otherwise $E_{ij}=0$. $A=\{a_{1},\ldots ,a_{N}\}$ denotes the attributes of *N* nodes. The scenario considered here is that one user has two accounts registered on two different social networks, and the two accounts are connected through an anchor link. Without loss of generality, we use one network as source network and the other as target network, denoted with $G^{s}$ and $G^{t}$ respectively. As shown in the Figure 1, some anchor links are already known between $G^{s}$ and $G^{t}$. For each node that has no anchor links in the source network $G^{s}$, the purpose of this paper is to find its corresponding node in the target network $G^{t}$. This can be formalized as the following anchor link prediction problem:

**Definition** **1.** *(**Anchor Link Prediction**) Given two attributed networks $G^{s}=\left(X^{s},E^{s},A^{s}\right)$ and $G^{t}=\left(X^{t},E^{t},A^{t}\right)$, and the existing anchor links $T=\{\left(x^{s},x^{t}\right)|x^{s}\in G^{s},x^{t}\in G^{t}\}$. The anchor link prediction problem is to predict potential anchor links across $G^{s}$ and $G^{t}$.*

As aforementioned in the introduction, our approach consists of two important components: one is the attributed network embedding and the other is the semi-supervised anchor link predictor. Next, we will introduce our APAN approach by elaborating them, respectively.

### 3.2. Learning Attributed Network Embedding

Skip-gram is a popular framework of embedding representation learning [14], which was first developed to capture the word semantic correlations. Given a word and its context $\left\{\left(x_{i},x_{c}\right)\right\}$, $x_{i}$ denotes the current word, and the context $x_{c}$ is a neighbor word around $x_{i}$ within a fixed window size. Skip-gram uses the embedding vector $e_{i}$ of word $x_{i}$ as input feature, and then predicts its context $x_{c}$ by minimizing the following log loss function:

(1) $$\begin{array}{cc} - & \sum_{\left(x_{i},x_{c}\right)}\log P\left(x_{c}|x_{i}\right)=-\sum_{\left(x_{i},x_{c}\right)}(w_{c}^{T}e_{i}-\log\sum_{c^{\prime }\in C}\exp\left(w_{c^{\prime }}^{T}e_{i}\right)) \end{array}$$

where C denotes the entire context space, which includes all the vocabularies of the corpus; ${\left\{w_{c}\right\}}_{c\in C}$ are the model parameters.

Inspired by Skip-gram, Perozzi et al. [10] developed DeepWalk to model the node correlation from the topology point of view. In DeepWalk, the embedding vector of a node is used to predict its network context, i.e., the node sequences generated by random walk regarding the node. Specifically, in each training node pair $\left(x_{i},x_{c}\right)$, $x_{i}$ is the current node, and the context $x_{c}$ is each of the neighboring nodes within a fixed window size regarding $x_{i}$ in the random-walk sequences. Here the context space C includes all the nodes in the network.

Next, we introduce how to extend the idea of DeepWalk for attributed network embedding. Let first assume that the embeddings for the source network $G^{s}$ and the target network $G^{t}$ are learnt independently here (In next subsection, we will discuss how to build connections between them). Suppose each node in the two networks is embedded into an *e*-dimensional vector.

To design the attributed network-embedding algorithm, we first need to understand the optimization strategy for Equation (1). A direct optimization to the objective is costly, because the second term must be normalized over the entire context space C, which is huge. In [14], a negative sampling strategy is used to tackle the problem. Specifically, the method re-casts the normalization-based optimization problem into a sampling-based binary classification problem. Assume $\left(x_{i},x_{c},\gamma \right)$ is a random sample drawn from a given probability distribution $P(x_{i},x_{c},\gamma )$. Here $x_{i}$ and $x_{c}$ represent the current node and a context, respectively. γ=+1 represents $\left(x_{i},x_{c}\right)$ is a positive pair, where $x_{c}$ is a node in the context of $x_{i}$. γ=−1 represents $\left(x_{i},x_{c}\right)$ is a negative pair that $x_{c}$ is not in the context of $x_{i}$. Given $\left(x_{i},x_{c},\gamma \right)$, we aim to minimize the cross-entropy loss to the binary class γ:

(2) $$-I\left(\gamma =1\right)\log\sigma \left(w_{c}^{T}e_{i}\right)-I\left(\gamma =-1\right)\log\sigma \left(-w_{c}^{T}e_{i}\right)$$

where σ is the sigmoid function, defined as $\sigma \left(x\right)=1/\left(1+e^{-x}\right)$; I(·) is the indicator function; when the argument is true it outputs 1, otherwise it outputs 0. As the samples follow the distribution $P(x_{i},x_{c},\gamma )$, the overall loss function can thus be expressed as:

(3) $$-E_{\left(x_{i},x_{c},\gamma \right)}\log\sigma \left(\gamma w_{c}^{T}e_{i}\right)$$

where *E* indicates the expectation operator.

In our attributed network-embedding scenario, the key issue now becomes generating samples with the distribution $P(x_{i},x_{c}\gamma )$. We give the concrete implementation in Algorithm 1. Two types of contexts are sampled in the algorithm. The first type is based on the network structure and the second type based on the node attributes *A*. By doing so, the learnt embeddings not only reflect the structure context, but also the attribute context.

**Algorithm 1** Sampling context algorithm**Input:** Network *G*, node attributes *A*, parameters $r_{1}$, $r_{2}$, *q*, *e* and *d*; **Output:**
$\left(x_{i},x_{c},\gamma \right)$;1:**if**$random_{1}<r_{1}$**then**2: γ←+1;3:**else**4: γ←−1;5:**end if**6:**if**$random_{2}<r_{2}$**then**7: Uniformly sample a random-walk sequence *S* of length *q*;8: **if** γ=+1 **then**9:  Under the condition |i−c|<d, sample $\left(x_{i},x_{c}\right)$ in *S*;10: **else**11:  Sample $x_{c}$ in C;12: **end if**13:**else**14: **if** γ=+1 **then**15:  Uniformly sample $\left(x_{i},x_{c}\right)$ which satisfies $a_{i}=a_{c}$;16: **else**17:  Uniformly sample $\left(x_{i},x_{c}\right)$ which satisfies $a_{i}\neq a_{c}$;18: **end if**19:**end if**

In the algorithm, we use a parameter $r_{1}\in \left(0,1\right)$ to control the proportion of positive and negative nodes, and use a parameter $r_{2}\in \left(0,1\right)$ to control the ratio of two types of contexts. As shown in lines 1∼5, we first determine whether to sample a positive sample or negative sample, in terms of $r_{1}$. Then in line 6, we generate a random number to determine whether to sample from the structure or the attribute context. If the number is smaller than $r_{2}$, structure context is chosen. We first produce a random-walk sequence *S* in line 7. If our previous decision is to sample a positive example, we produce the context $x_{c}$ such that it is within the window size of *d* regarding $x_{i}$ (lines 8∼10), otherwise we randomly choose an example from C (lines 10∼12). When sampling attribute context (lines 13∼19), positive examples are randomly chosen from the nodes that have the same attribute values, while negative examples are from the ones that have different attribute values.

### 3.3. Semi-Supervised Anchor Link Prediction

In the subsection, we introduce how to use the observed anchor links for the embedding learning. Given a potential anchor link pair $(x_{l}^{s},x_{n}^{t})\in T$ and the corresponding embedding vectors $e_{l}^{s}$ and $e_{n}^{t}$, the probability that the anchor link exists can be expressed as:

(4) $$P\left(x_{l}^{s},x_{n}^{t}\right)=\sigma \left({e_{l}^{s}}^{T}\cdot e_{n}^{t}\right)=1/\left(1+e^{-{e_{l}^{s}}^{T}\cdot e_{n}^{t}}\right)$$

where σ is sigmoid function. To capture more complex associations, we can build a *k* layers feed-forward neural network as our predictor. The *k*-th layer $h^{k}$ of the neural network is a nonlinear function of the previous hidden layer $h^{k-1}$, defined as

(5) $$h^{k}\left(e\right)=ReLU\left(W^{k}h^{k-1}\left(e\right)+b^{k}\right)$$

where ReLU(x)=max(x,0), $W^{k}$ and $b^{k}$ are parameters of *k*-th layer, and $h^{0}\left(e\right)=e$. By using the complex predictor, Equation (4) is rewritten as:

(6) $$\begin{array}{cc} P(x_{l}^{s},x_{n}^{t}) & =\sigma (h^{k}{\left(e_{l}^{s}\right)}^{T}\cdot h^{k}\left(e_{n}^{t}\right))\\ & =1/(1+e^{-h^{k}{\left(e_{l}^{s}\right)}^{T}\cdot h^{k}\left(e_{n}^{t}\right)}) \end{array}$$

**Figure 2:**
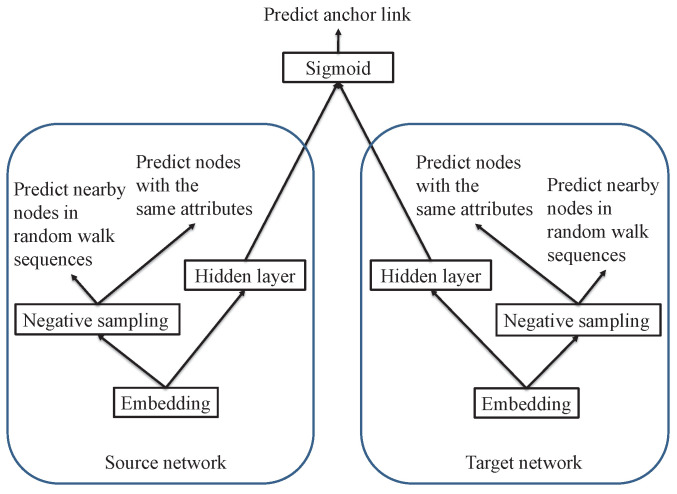
APAN network architecture.

Combining Equation (6) with Equation (3), we obtain the following objective function for the anchor link prediction problem:

(7) $$\begin{array}{cc} - & \sum_{(x_{l}^{s},x_{n}^{t})\in T}\log P\left(x_{l}^{s},x_{n}^{t}\right)-\lambda_{1}E_{\left\{\left(x_{i}^{s},x_{c}^{s},\gamma \right)|x_{i}^{s},x_{c}^{s}\in G^{s}\right\}}\log\sigma \left(\gamma {w_{c}^{s}}^{T}e_{i}^{s}\right)\\ & -\lambda_{2}E_{\left\{\left(x_{i}^{t},x_{c}^{t},\gamma \right)|x_{i}^{t},x_{c}^{t}\in G^{t}\right\}}\log\sigma \left(\gamma {w_{c}^{t}}^{T}e_{i}^{t}\right) \end{array}$$

where $\lambda_{1}$ and $\lambda_{2}$ are two parameters. In Equation (7), the first item is the loss of anchor link prediction, the second item is the loss of context predictions in source network $G^{s}$, and the third item is the loss of context predictions in target network $G^{t}$. The network structure of APAN algorithm is shown in Figure 2.

By optimizing Equation (7), we ultimately obtain the embedding vectors e of all nodes in the source network $G^{s}$ and the target network $G^{t}$. When predicting anchor links, given a node $x_{l}^{s}$ in the source network $G^{s}$, we can calculate the probabilities that $x_{l}^{s}$ has anchor links with all the nodes in the target network $G^{t}$, by using Equation (6). Sorting them in terms of the probabilities offers us a list of potential anchor links.

When training the proposed APAN, a stochastic gradient descent in the mini-batch mode is adopted [24]. In each iteration, a set of node pairs in the anchor links set *T* is first sampled and a gradient calculation is performed to optimize the loss of anchor link prediction. Subsequently, we sample a set of context $\left(x_{i}^{s},x_{c}^{s},\gamma \right)$ in the source network $G^{s}$ and perform a gradient calculation to optimize the loss function of predicting context in $G^{s}$. Similarly, we sample a set of context $\left(x_{i}^{t},x_{c}^{t},\gamma \right)$ in the target network $G^{t}$ and perform a gradient calculation to optimize the loss of predicting context in $G^{t}$. The model training procedure is implemented as Algorithm 2.

**Algorithm 2** Model training**Require:** Attributed networks $G^{s}$ and $G^{t}$, parameters $\lambda_{1}$ and $\lambda_{2}$, batch sizes $K_{1}$, $K_{2}$ and $K_{3}$;
1:
**for** it=1→Max_It **do**
2: Sample a group of node pairs of size $K_{1}$ in the anchor links set *T*;3: Let $R_{1}=-\frac{1}{K_{1}}\sum_{\left(x_{l}^{s},x_{n}^{t}\right)}\log P\left(x_{l}^{s},x_{n}^{t}\right)$, perform a gradient calculation on $R_{1}$;4: Sample a group of contexts $\left(x_{i}^{s},x_{c}^{s},\gamma \right)$ of size $K_{2}$ in the source network $G^{s}$;5: Let $R_{2}=-\frac{\lambda_{1}}{K_{2}}\sum_{\left(x_{i}^{s},x_{c}^{s},\gamma \right)}\log\sigma \left(\gamma {w_{c}^{s}}^{T}e_{i}^{s}\right)$, perform a gradient calculation on $R_{2}$;6: Sample a group of contexts $\left(x_{i}^{t},x_{c}^{t},\gamma \right)$ of size $K_{3}$ in the target network $G^{t}$;7: Let $R_{3}=-\frac{\lambda_{2}}{K_{3}}\sum_{\left(x_{i}^{t},x_{c}^{t},\gamma \right)}\log\sigma \left(\gamma {w_{c}^{t}}^{T}e_{i}^{t}\right)$, perform a gradient calculation on $R_{3}$.8:
**end for**



## 4. Experiments

In this section, we conduct experiments to compare the proposed APAN algorithm with state-of-the-art techniques.

### 4.1. Datasets and Baselines

In the experiments, we use three real-world attributed networks, which are Flickr and Lastfm datasets from [25], and Douban dataset from [26]. Following [23], we adopt the following ways to construct our datasets (Table 1).

**Flickr vs. Lastfm**. We extract the subnetworks from Flickr and Lastfm, which contain 4935 nodes and 4496 nodes, respectively. The edges in the two networks are who-follow-whom relationship. We consider the gender of a user as node attribute. For the users whose gender information is missing, we fill in the values of ’unknown’.

**Douban Online vs. Douban Offline**. The offline network is constructed according to users’ co-occurrence in social gatherings. There is an edge in the offline network between two users if they participate in the same offline events more than ten times. The constructed offline network includes 1118 users and we extract a subnetwork with 3906 nodes from the provided online network that contains all these offline users. We treat the location of a user as the node attribute.

We compare APAN algorithm with the following baselines:

- PALE [13]: This algorithm is a network-embedding-based anchor link prediction algorithm. PALE employs network embedding with awareness of observed anchor links as supervised information to capture the structural regularities and further learns a stable cross-network mapping for anchor link prediction.
- ULink [27]: ULink is a projection algorithm designed based on latent user space modelling. They build the latent user space through projection matrix.
- FINAL [23]: FINAL is proposed to solve the attributed network alignment problem. It leverages the node attribute information to guide (topology-based) alignment process.
- APAN-N: This algorithm is a variant of our proposed APAN algorithm. When predicting context using negative sampling, APAN-N only predicts context based on network structure and does not use nodes’ attributes.

In the comparison, we implemented the PALE method and use the original implementations of ULink and FINAL methods.

For the anchor link prediction problem, the widely used evaluation metric is to compare the top-*k* ranking list of a potential matching account. The higher the rank of the ground-truth account in the list, the better. In this paper, we evaluate all methods by computing top-*k* precision [27] for each test user as follows:

(8) $$h\left(x\right)=\frac{k-(hit(x)-1)}{k}$$

where hit(x) represents the position of ground-truth account in the returned top-k users. We report the average precision of all the tested users $x_{i}$ as the result: $\sum_{i=1}^{N}h\left(x_{i}\right)/N$, which is denoted by “Hit-precision”.

In our experiments, we randomly partition the ground-truth anchor links into five groups and conduct five-fold cross-validation and report the average results. We set the model parameters to $r_{1}=2/3$, q=10, e=50, d=3, $K_{1}=1000$, $K_{2}=2000$ and $K_{3}=2000$. We found that our model is not very sensitive to these parameters. We tune $r_{2}$, $\lambda_{1}$ and $\lambda_{2}$ via cross-validation method.

### 4.2. Experimental Results

**Figure 3:**
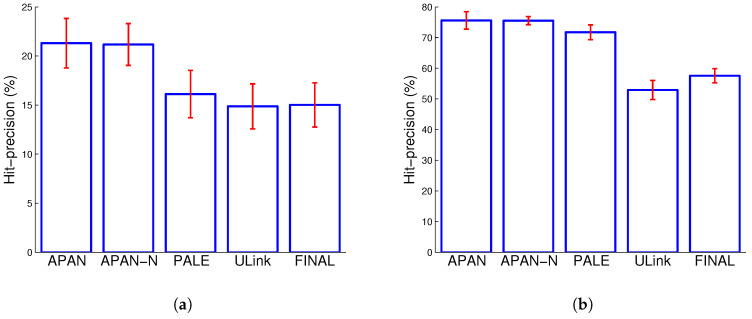
The average Hit-precision@*k* performance on real datasets. (**a**) Flickr-Lastfm networks; (**b**) Douban online-offline networks.

**Figure 4:**
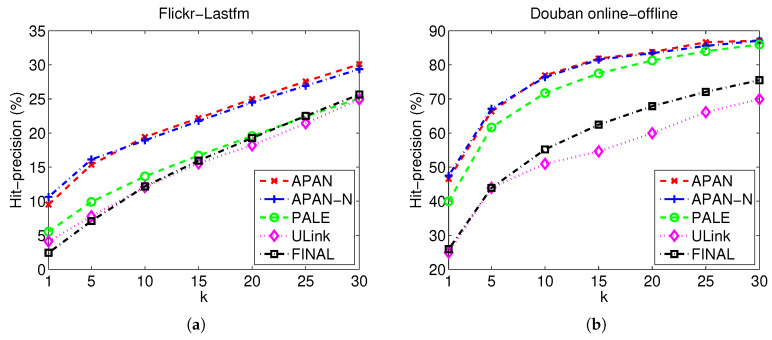
Hit-precision@*k* performance on real datasets. (**a**) Flickr-Lastfm networks; (**b**) Douban online-offline networks.

Table 2, Figure 3 and Figure 4 show the Hit-precision@*k* results of different compared algorithms with different *k*. From the figures, we can easily judge the performance trend when varying the number labeled data, whereas the detailed performed can be easily observed from the table. As can be seen from the experimental results, APAN and APAN-N achieve better performance than the baseline methods in most cases. Specifically, APAN outperforms PALE, ULink and FINAL by more than 6%. For instance, the Hit-precision of APAN is 19.42% while the result of PALE is 13.65% when *k* equals to 10 for Flickr-Lastfm networks; the Hit-precision of APAN is 46.53% while the result of PALE is 39.98% when *k* equals to 1 for Douban online-offline networks. Moreover, we observe that APAN which uses node attributes yields better performance than APAN-N that leverages only the network structure. The observation suggests that APAN can effectively exploit network structure and node attributes. This implies that the node attributes and network structure contain useful information to give a comprehensive view about the user. An effective model for network data should thus consider both the node attributes and network structure in the anchor link prediction task. Also, we find that the results of APAN and APAN-N are very close. This is because there are many missing values in the node attributes. In the datasets, the gender and the location are used as attributes of the nodes. For the users whose attributes information are missing, we fill in the values of ’unknown’. The loss of attribute information degenerates the performance of APAN method. We find that network-embedding-based methods APAN, APAN-N, and PALE deliver better results than the other methods. In particular, an accuracy improvement of 17% against other algorithms is observed when k=5 in Douban online-offline networks (PALE with Hit-precision 61.66% versus ULink with 43.93%). The observation demonstrates the effectiveness and merits of network-embedding methods. Compared to the conventional approaches, network-embedding-based methods represent each node into a continuous real-value vector. By doing so, the network structure regularities and attribute information can be summarized into the vector, which is better than the hand-crafted features in conventional methods.

**Figure 5:**
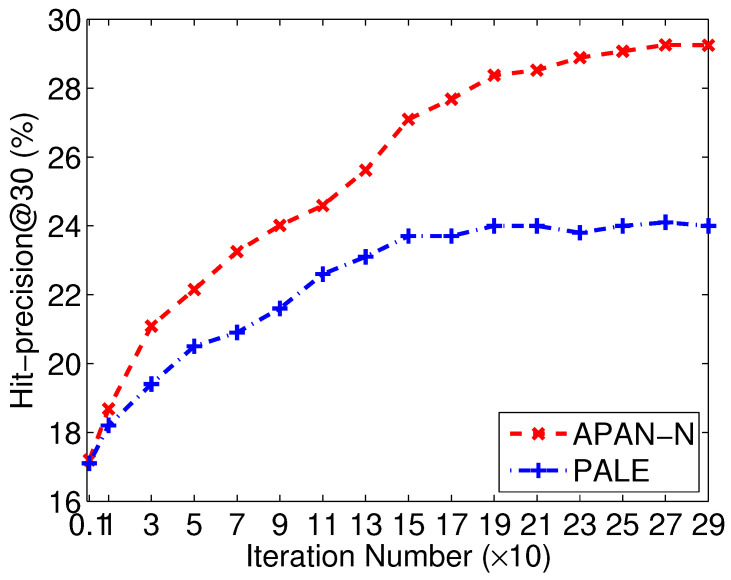
Comparison of APAN-N and PALE in different iterations on Flickr-Lastfm networks.

Next, we compare APAN-N and PALE algorithms, which are all based on network-embedding learning and use only network structure. We can see APAN-N performs better than PALE in most cases. APAN-N achieves an accuracy improvement of 6% against PALE when *k* equals to 5 for Douban online-offline networks. There are two main reasons. On the one hand, the proposed APAN-N works in a semi-supervised manner. During the training phase, three objectives, namely, the anchor link prediction, the context prediction in the source network and the one in the target network, are iterated. Hence, the produced node embeddings incorporate both the supervised and unsupervised information. However, PALE breaks up the network-embedding learning and exist anchor link prediction into two independent phases. As a result, the node embedding vectors produced by PALE are only related to the network structure. Hence, the embeddings produced by our APAN-N are more helpful for the anchor link prediction task. On the other hand, PALE uses the first-order proximity structure in the network-embedding learning process [11]. The method only models the local adjacency of each node, but ignores the global connection property in the network. Therefore, PALE is not sufficient to preserve the intrinsic structure regularities of networks. Instead, our APAN-N uses the truncated random-walk sequences to learn the embeddings, which can capture both the local and global structure properties. This can also be verified by Figure 5, which depicts the performance changes of APAN-N and PALE. A big gap can be found as the number of iterations increases. Due to the reasons, APAN-N works better than PALE.

### 4.3. Parameter Study

In this subsection, we investigate how different values of the parameter $r_{2}$ and the dimension of the embedding vectors affect the performance of APAN.

**Figure 6:**
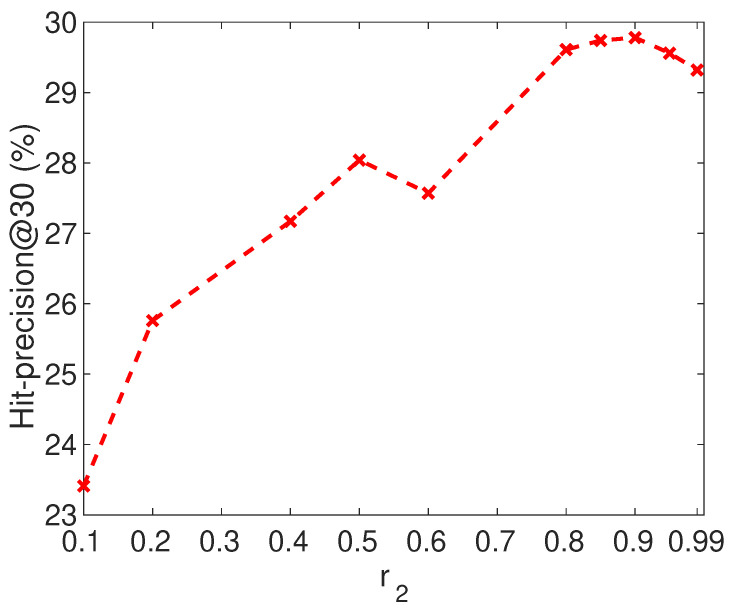
The tuning of $r_{2}$ on Flickr-Lastfm networks.

For our proposed APAN method, we use parameter $r_{2}$ to control the ratio of two types of contexts. The larger $r_{2}$ is, the more important network structure is. Figure 6 shows the Hit-precision@30 of APAN using different values of parameter $r_{2}$ on Flickr-Lastfm networks. From the figure, we observe that the accuracy is very low when $r_{2}$ is small, and the accuracy increases when $r_{2}$ becomes large. It achieves good performance with the $r_{2}$ varying from 0.8 to 0.9. The large value of $r_{2}$ indicates the importance of network structure. In the datasets, the node attribute includes the gender and the location. Since there are many missing values in the attributes, and these two kinds of attribute are not strong enough to link users, the structural information is more discriminative than the attribute information.

**Figure 7:**
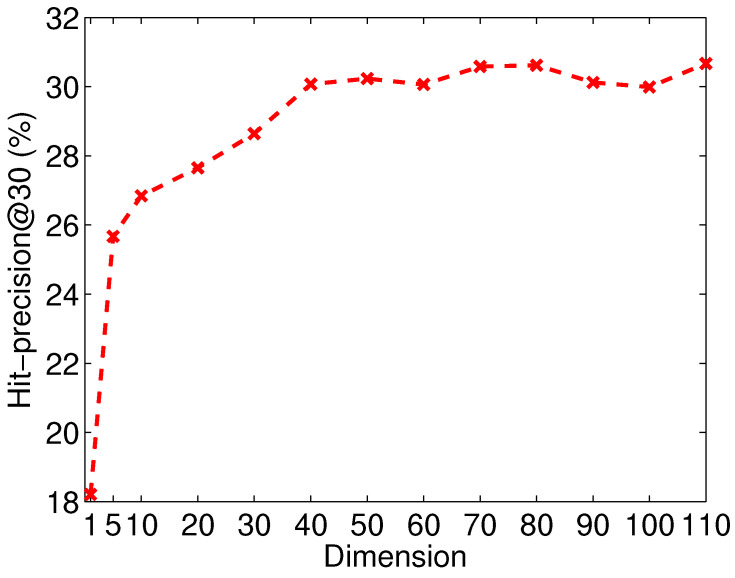
Tuning the dimension of the embedding vectors on Flickr-Lastfm networks.

We investigate the sensitivity of the dimension of the embedding vectors. Figure 7 shows the Hit-precision@30 of APAN with various dimensions on Flickr-Lastfm networks. We observe the performance is poor when the dimensionality is under 30. APAN reaches a relatively stable and promising performance after the dimensionality is higher than 50. This indicates that APAN model is robust with the tuning of dimensions.

## 5. Conclusions

In this paper, we propose a novel semi-supervised network-embedding model (APAN) to tackle the anchor link prediction across attributed networks. APAN represents each node (user) of the multiple networks by a low-dimensional vector, which is learnt with awareness of observed anchor links as semi-supervised information, and topology structure and attributes as input. By doing so, the nodes that have similar structure contexts and attributed information will have similar embedding vectors. Also, the anchor link predictor is simultaneously trained as the embeddings are learnt. The real-world data sets are used to evaluate the performance of the proposed APAN model. Experimental results show that APAN outperforms state-of-the-art competitors.

APAN has two limitations. Firstly, since the node embedding vectors produced by APAN are related to the network structure, the accuracy will be low when the topology structures of the two networks are widely distinct. Secondly, social networks are dynamically changing over time. The APAN method cannot extract features dynamically. Our next work will solve the above two problems, we may consider integrating more types of information, such as the temporal information, into APAN so that the method can be more robust, and develop a dynamic anchor link prediction algorithm to take advantage of incremental data for improving the performance.

## Figures and Tables

**Table 1:** Statistics of the datasets.

Datasets	#Nodes	#Edges
Flickr	4935	15,884
Lastfm	4496	10,628
Douban Online	3906	16,328
Douban Offline	1118	3022

**Table 2:** Performance of different models on real datasets (%), boldface indicates the best performance.

Dataset	Evaluation Metric	APAN	APAN-N	PALE	ULink	FINAL
	Hit-p@1	9.51 ± 2.82	**10.63 ± 1.17**	5.53 ± 1.57	4.10 ± 1.22	2.43 ± 0.90
	Hit-p@5	15.35 ± 2.58	**16.12 ± 2.25**	9.91 ± 2.73	7.79 ± 2.06	7.10 ± 0.60
	Hit-p@10	**19.42 ± 2.73**	18.93 ± 2.37	13.65 ± 3.00	12.07 ± 1.92	12.18 ± 1.76
Flickr-Lastfm	Hit-p@15	**22.18 ± 2.70**	21.75 ± 2.41	16.70 ± 2.81	15.55 ± 3.24	15.95 ± 2.45
	Hit-p@20	**24.98 ± 2.57**	24.48 ± 2.41	19.57 ± 2.51	18.20 ± 2.91	19.28 ± 3.04
	Hit-p@25	**27.57 ± 2.25**	26.92 ± 2.23	22.42 ± 2.20	21.42 ± 2.88	22.51 ± 3.28
	Hit-p@30	**30.09 ± 1.94**	29.36 ± 2.04	25.08 ± 2.07	24.93 ± 1.81	25.65 ± 3.63
	Hit-p@1	46.53 ± 2.80	**47.49 ± 2.98**	39.98 ± 3.30	24.95 ± 3.05	25.93 ± 2.17
	Hit-p@5	66.31 ± 3.23	**67.12 ± 1.29**	61.66 ± 2.04	43.93 ± 4.03	43.90 ± 1.20
	Hit-p@10	**76.91 ± 3.09**	76.38 ± 0.95	71.72 ± 2.06	50.95 ± 2.74	55.19 ± 2.27
Douban online-offline	Hit-p@15	**81.84 ± 2.79**	81.54 ± 0.98	77.49 ± 2.24	54.62 ± 1.85	62.44 ± 2.81
	Hit-p@20	**83.76 ± 2.39**	83.43 ± 1.00	81.25 ± 2.24	59.94 ± 2.36	67.86 ± 2.77
	Hit-p@25	**86.62 ± 2.12**	85.55 ± 1.12	83.99 ± 2.17	66.11 ± 3.22	72.08 ± 2.64
	Hit-p@30	**87.12 ± 1.99**	87.09 ± 1.03	85.98 ± 2.14	69.92 ± 4.39	75.49 ± 2.34
